# Patient and Healthcare Professional Perspectives on Crohn’s Perianal Fistula Treatment: Results From a Discrete Choice Experiment

**DOI:** 10.1093/crocol/otae076

**Published:** 2025-01-04

**Authors:** Jeanne Jiang, Bridgett Goodwin, Amod Athavale, Susan E Cazzetta, Lily Chen, Josiah Edelblut, Tao Fan, Nandini Hadker, Pradeep P Nazarey

**Affiliations:** GI Medical, Takeda Pharmaceuticals USA, Inc., Lexington, MA, USA; GI Medical, Takeda Pharmaceuticals USA, Inc., Lexington, MA, USA; Evidence Strategy, Trinity Life Sciences, Waltham, MA, USA; GI Medical, Takeda Pharmaceuticals USA, Inc., Lexington, MA, USA; GI Medical, Takeda Pharmaceuticals USA, Inc., Lexington, MA, USA; Evidence Strategy, Trinity Life Sciences, Waltham, MA, USA; GI Medical, Takeda Pharmaceuticals USA, Inc., Lexington, MA, USA; Evidence Strategy, Trinity Life Sciences, Waltham, MA, USA; GI Medical, Takeda Pharmaceuticals USA, Inc., Lexington, MA, USA

**Keywords:** : Crohn’s perianal fistulas, discrete choice experiment, CPF-related procedures/surgeries, treatment attribute importance, stated preference methodology

## Abstract

**Background:**

Crohn’s perianal fistulas (CPF) are difficult to manage and often require multiple interventions. This study aimed to assess the preferences of patients and healthcare professionals (HCPs) for attributes of CPF-related procedures/surgeries to better inform CPF management.

**Methods:**

This US cross-sectional, observational study was conducted via a web-enabled questionnaire (October 2021-January 2022) among patients aged 21-89 years with a self-reported physician diagnosis of CPF (with or without CPF-related surgery experience) and HCPs (gastroenterologists and colorectal surgeons who managed ≥3 patients with CPF in the past 12 months). Patient and HCP preferences for CPF-related procedure/surgery attributes were assessed using a discrete choice experiment and stated preference methodology.

**Results:**

In total, 100 patients and 137 HCPs were recruited. Benefits of therapy (symptom control and/or fistula closure) were rated as the most important CPF treatment attribute by both patients and HCPs influencing treatment decisions (mean relative importance 23.9 and 36.3, respectively). The mean relative importance of procedure invasiveness and postoperative discomfort was higher for patients (19.3 and 20.2, respectively) than for HCPs (14.3 and 11.0, respectively), whereas the mean relative importance of fecal incontinence was greater for HCPs than patients (25.0 vs. 19.3, respectively).

**Conclusions:**

Patients and HCPs have different perspectives on the importance of specific CPF-related procedure/surgery attributes. The attributes identified as important to patients and HCPs in this study should be considered when managing patients with CPF and making treatment decisions.

## Introduction

Crohn’s disease (CD) is a chronic inflammatory disorder of the gastrointestinal tract.^1^ Crohn’s perianal fistulas (CPF) are one of the most common and disabling complications of CD, affecting 20-40% of patients with CD.^2^ The most common CPF symptoms include pain, perianal swelling and itching, fecal incontinence (FI), and discharge of pus, feces, or blood from cutaneous fistula tracts, which can negatively impact a patient’s health-related quality of life (HRQoL).^3–6^

CPF can be challenging to manage, and a combination of pharmacotherapy with surgical procedures and a multidisciplinary team (MDT) approach is usually required for long-term treatment.^5,7–9^ Despite the use of current CPF pharmacotherapies and procedures/surgeries, treatment failure and relapse are common, and patients often need to undergo repeated cycles of interventions and surgeries.^5,9^

Previous studies have shown that patients and healthcare professionals (HCPs) differ in their treatment goals and in recognizing important HRQoL factors associated with anal fistulas and their treatment.^10^ Gaining a better understanding of the perspectives of patients and HCPs on CPF treatment options is required to help HCPs with CPF management, treatment decisions, and to improve patient HRQoL.

This study assessed patient and HCP preferences for attributes of CPF-related procedures/surgeries using a discrete choice experiment (DCE) and stated preference methodology. DCE methodology has been increasingly used in healthcare research as it can quantitatively assess the strength of preferences for medical interventions, thereby providing an accurate assessment of patient preferences for all potential therapy options and their attributes.^11,12^ DCEs mimic choice behavior as they offer the participants the opportunity to evaluate trade-offs among various attributes in a clinically realistic, although hypothetical, context. In addition, by simulating a range of possible scenarios and offering participants a range of alternative choices during the treatment decision-making, DCEs can “hide” a treatment attribute of interest within a pool of potential trade-offs, thereby minimizing biases that could be introduced if the importance of a particular treatment attribute was emphasized.^12^ Finally, these tools make for an attractive option as the questions are easy to understand and the results easy to interpret.^12^ Therefore, DCEs can be useful for evaluating which attributes of CPF-related procedures/surgeries matter most to patients and HCPs when making treatment decisions.

To our knowledge, this study includes the first DCE conducted in a US population of patients with CPF to evaluate treatment preferences.

## Methods

### Study Design

This cross-sectional, observational study was conducted among US adults with CPF and HCPs (gastroenterologists [GEs] and colorectal surgeons [CRSs]) who manage patients with CPF. The study was conducted via a 30-minute web-enabled questionnaire shared by Dynata LLC (NY, USA) from October 2021 to December 2021 for the patient survey and October 2021 to January 2022 for the HCP survey.

Patients were invited to participate in the study based on self-reported profile data for CPF as part of Dynata LLC’s patient panel. HCPs were invited to participate in the study based on medical specialty and focus of practice as part of Dynata LLC’s HCP panel. The web-enabled questionnaire was pretested by conducting 45-minute telephone cognitive interviews with patients with CPF (*n* = 4) and HCPs who treat patients with CPF (*n* = 4). During these pretest interviews, the interviewer hosted the web-enabled questionnaire on their computer and shared their screen with the patients and HCPs via a screen sharing software. The purpose of these interviews was to assess comprehension of the questions as intended and to identify any potential sources of response error. Upon successful completion of the interview, patients and HCPs were provided with a gift card or check from Dynata LLC (valued at $100 and $250, respectively) as compensation for their time and participation within ~3 weeks of completing their interview. These patients/HCPs did not go on to participate in the web-enabled questionnaire (with the exception of CRSs due to difficulties in recruiting from this specialty). Patients and HCPs who participated in the web-enabled questionnaire were also compensated by Dynata LLC with a voucher or check (valued at $30 and $80, respectively). Compensation payments to patients and HCPs were approved by the Institutional Review Board (IRB) in line with the Pharmaceutical Research and Manufacturers of America (PhRMA) Principles on Conduct of Clinical Trials and PhRMA Code on Interactions with HCPs.^13,14^

### Study Population

#### Patients

Patients aged 21-89 years were eligible for inclusion if they had a self-reported physician diagnosis of CPF and had been treated for CPF (including pharmacotherapy and/or seton placement, with or without any other CPF-related procedure/surgery) in the past 12 months. Patients with a diagnosis of ulcerative colitis were excluded. Given the expected issues with recruiting patients with CPF and the exploratory nature of this study, a sample of ~100 patients with CPF was deemed feasible based on the initial field assessments. A minimum sample size of 99 patients was deemed appropriate assuming a DCE design with a maximum of 6 treatment attributes with up to 4 levels each and an opt-out option.^15^

#### HCPs

HCPs were eligible for inclusion if they were board certified or eligible for board certification in gastroenterology or colorectal surgery, had been in practice for 3-35 years postresidency, spent ≥70% of professional time treating patients at the time of the survey, treated at least 50 different patients with any health condition in an average month, and treated at least 20 different patients with CD in an average month. In addition, GEs were eligible if they had treated at least 3 adult patients with CPF in the past 12 months, and CRSs were eligible if they had performed 3 or more CPF-related procedures or surgeries in the past 12 months. HCPs were excluded if they were affiliated with a pharmaceutical manufacturer, contract research organization, medical equipment manufacturer, market research or advertising firm, a government agency, or the US Food and Drug Administration, or had participated in market research for CPF in the past month. HCP sample size was based on sufficient numbers needed to conduct subanalyses of specialty groups.

### Study Objectives

The objective of this study was to assess patient and HCP preferences for CPF-related procedures/surgeries using a DCE and a stated preference methodology.

### Study Measures

Patient demographics and clinical characteristics, and HCP clinigraphics and HCP-reported patient characteristics were collected. Patients were asked to report clinical characteristics (eg, severity of CPF) as reported to them by their physician.

Patients were asked to report their experience with CPF-related procedures/surgeries and their surgery complication experience.

**Figure 1.**
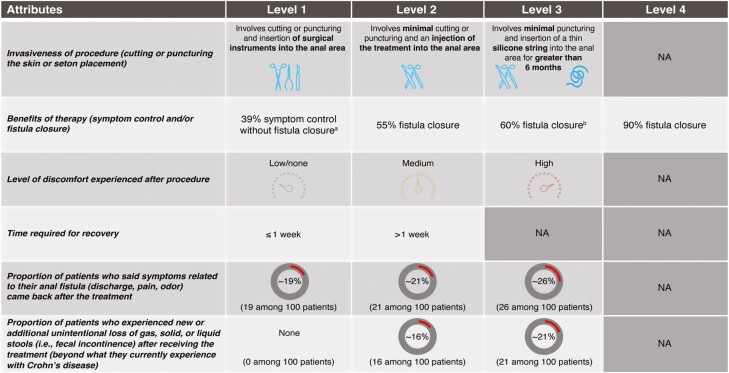
CPF-related procedure/surgery attributes and levels used to generate hypothetical surgical treatment options in the DCE to determine patient preferences. ^a^This level was only shown with Attribute 1, Level 3; ^b^No level with fistula closure was shown with seton level above. CPF, Crohn’s perianal fistulas; DCE, discrete choice experiment; NA, not applicable.

A DCE was used to assess the attributes driving CPF-related procedure/surgery preferences. Patients and HCPs were asked to evaluate 2 hypothetical procedure/surgery profiles at a time and to identify their preferred procedure/surgery of the 2 or select neither. These procedures/surgeries included 6 attributes: (1) invasiveness of the procedure, (2) benefits of therapy (symptom control and/or fistula closure), (3) level of discomfort experienced after procedure, (4) time required for recovery, (5) recurrence rate, and (6) FI rate. The levels for each attribute (2-4 levels), based on evidence in currently available literature, were used to create 14 sets of 2 hypothetical treatment profiles (Figure 1 and Supplementary Figure 1).^16–20^

### Statistical Analysis

Demographics, clinical characteristics, and treatment experience data were summarized using descriptive statistics and Q Research Software 5.9.7.0.

Data collected from the DCE were analyzed using a hierarchical Bayesian model and Lighthouse Studio 9.10.1 software using the attribute levels as predictor variables and choice as the outcome variable. The model generated mean relative attribute importance scores for each attribute and mean relative preference weights (RPWs) for each level within the attributes tested.

An overall mean relative importance score was used as a measure of how important an attribute is in selecting a procedure/surgery for CPF, in the context of all attributes tested in the DCE design. The mean relative importance was represented by a normalized score: in total, 100 points were allocated across each product attribute from the DCE based on the importance of each attribute when making CPF treatment decisions for both HCPs and patients. For the 6 attributes, if all were deemed equally important, then each would receive a point allocation of 16.7. Therefore, attributes perceived as important to respondents had a score of ≥16.7.

Mean RPWs were used as a measure of preference for an attribute level, relative to any other attribute level, in the context of all attribute levels tested in the DCE design. A high value indicates a higher preference for that attribute level in selecting a treatment/procedure for CPF and a lower value indicates a lower preference.

## Results

### Study Population

#### Patients

In total, 100 patients were recruited, 50 with CPF-related surgery experience and 50 without CPF-related surgery experience (other than seton placement) (Table 1A). The majority of patients (73/100, 73%) reported CPF severity as moderate or severe (as classified by a physician), more than half of patients (53/100, 53%) had complex fistulas, and more than half of patients (52/100, 52%) reported fistula recurrence. The mean (standard deviation [SD]) number of symptoms experienced per patient was 4.9 (2.7). The majority of patients with CPF (59/100, 59%) had experienced FI, which was resolved for 41/59 (69%) of those patients. Of those patients currently experiencing FI, the majority (83%) experienced FI on a weekly or monthly basis (Table 1A).

**Table 1.** (A) Patient demographics and clinical characteristics. (B) HCP clinigraphics and HCP-reported patient characteristics.

(A) Patient demographics and clinical characteristics	
Characteristic	All patients *N* = 100
**Age**, mean (SD)	40.0 (12.2)
**Years since CPF diagnosis**, mean (SD)	9.1 (9.9)
**Sex**, *n* (%)	
Male	44 (44)
**Race,** *n* (%)	
White	88 (88)
Black or African American	10 (10)
Other/Prefer not to answer	2 (2)
**Ethnicity**, *n* (%)	
Non-Hispanic	86 (86)
Hispanic	12 (12)
Unknown/Prefer not to answer	2 (2)
**Unique CPF**, mean (SD)	2.7 (2.4)
**CPF severity (classified by a physician)**, *n* (%)	
Mild	15 (15)
Moderate	45 (45)
Severe	28 (28)
Do not know/my doctor has not told me the level of severity	12 (12)
**Complex CPF**, *n* (%)	
Yes	53 (53)
No	28 (28)
I do not know	19 (19)
**Status of fistula**, *n* (%)	
A fistula that is active, draining, and is currently being treated	52 (52)
A fistula that is active but not draining and is currently being treated	53 (53)
A fistula that is still forming and is untreated	18 (18)
**Fistula recurrence**, *n* (%)	
Yes	52 (52)
No	48 (48)
**Number of symptoms experienced**, mean (SD)	4.9 (2.7)
**Ever experienced FI (*n* = 100)**, *n* (%)	
Yes	59 (59)
No	41 (41)
**FI resolved (*n* = 59)** ^a^, *n* (%)	
Yes	41 (69)
No	18 (31)
**Impact of FI on QoL (*n* = 59)** ^a^, mean (SD)	6.8 (2.3)
**Frequency of FI (*n* = 18)** ^b^, *n* (%)	
Daily	3 (17)
Weekly	8 (44)
Monthly	7 (39)
Yearly	0 (0)
(B) HCP clinigraphics and HCP-reported patient characteristics	
Characteristic	All HCPs *N* = 137
**Sex**, *n* (%)	
Male	105 (77)
**Years in active clinical practice**, mean (SD)	15.6 (7.8)
**Practice setting type**, *n* (%)	
Private practice	59 (43)
Community hospital	37 (27)
Academic hospital	30 (22)
University hospital	11 (8)
**Practice setting location**, *n* (%)	
Urban	63 (46)
Suburban	67 (49)
Rural	7 (5)
**Patients with CD**, mean (SD)	110.8 (76.0)
**Patients with CPF**, mean (SD)	29.4 (16.7)
**Procedures and surgeries performed**, mean (SD)	21.8 (13.4)
**Patients by CPF severity**, %	
Mild	30
Moderate	41
Severe	28
**Patient by lines of therapy**, %	
1L	45
2L	33
3L+	22

Abbreviations: CD, Crohn’s disease; CPF, Crohn’s perianal fistulas; FI, fecal incontinence; HCP, healthcare professional; QoL, quality of life; SD, standard deviation.

^a^Patients ever experienced FI;

^b^Patients currently experiencing FI.

#### HCPs

In total, 137 HCPs were recruited (GEs: 77/137 [56%]; CRSs: 60/137 [44%]). The mean (SD) number of years in active practice (postresidency) was 15.6 (7.8) and 59/137 (43%) of HCPs worked in private practice. The majority (69%) of the patients managed by HCPs had moderate or severe CPF (Table 1B).

### Study Measures

#### Treatment experience

The majority of patients with CPF-related surgery experience had a fistulectomy/fistulotomy (29/50, 58%) and/or endorectal/anal advancement flap (26/50, 52%). The failure rate for CPF-related procedures and surgeries ranged from 1/16 (6%) for anal fistula plug to 5/24 (21%) for long-term seton placement (Table 2).

**Table 2.** CPF-related procedures/surgeries.

Characteristic	All patients *N *= 100
**Experience with CPF-related procedures/surgeries in the past 12 months**, *n* (%)	
Long-term seton placement^a^	24 (24)
% failure rate	5 (21)
Short-term seton placement^a^	30 (30)
% failure rate	6 (20)
Endorectal/anal advancement flap^b^	26 (52)
% failure rate	3 (12)
Fibrin glue^b^	14 (28)
% failure rate	2 (14)
Anal fistula plug^b^	16 (32)
% failure rate	1 (6)
Fistulectomy/fistulotomy^b^	29 (58)
% failure rate	2 (7)
LIFT^b^	13 (26)
% failure rate	1 (8)
**Frequency of CPF-related procedures/surgeries in the past 12 months**, mean (SD)	7.5 (8.6)
**Patients who experienced CPF-related procedure/surgery/seton placement in the past 12 months**, *n* (%)	
One procedure/surgery	24 (24)
Two or more procedures/surgeries	46 (46)
**Number of complications post CPF-related surgeries**, mean (SD)	2.8 (2.1)
**Experience with any complication(s) post a procedure/surgery** ^c^, *n* (%)	
Worsening of pain/difficulty with bowel movements	23 (33)
Worsening of pain and swelling around the anus	21 (30)
Fever/infection	20 (29)
Development of additional fistula or fistula worsening	19 (27)
Worsening of irritation of the skin around the anus from drainage	18 (26)

Abbreviations: CPF, Crohn’s perianal fistulas; LIFT, ligation of the intersphincteric fistula tract; SD, standard deviation.

^a^Patients with and without CPF-related surgery experience;

^b^Only patients with CPF-related surgery experience;

^c^Complications in more than 25% of all patients.

#### DCE: Patient preferences

**Figure 2.**
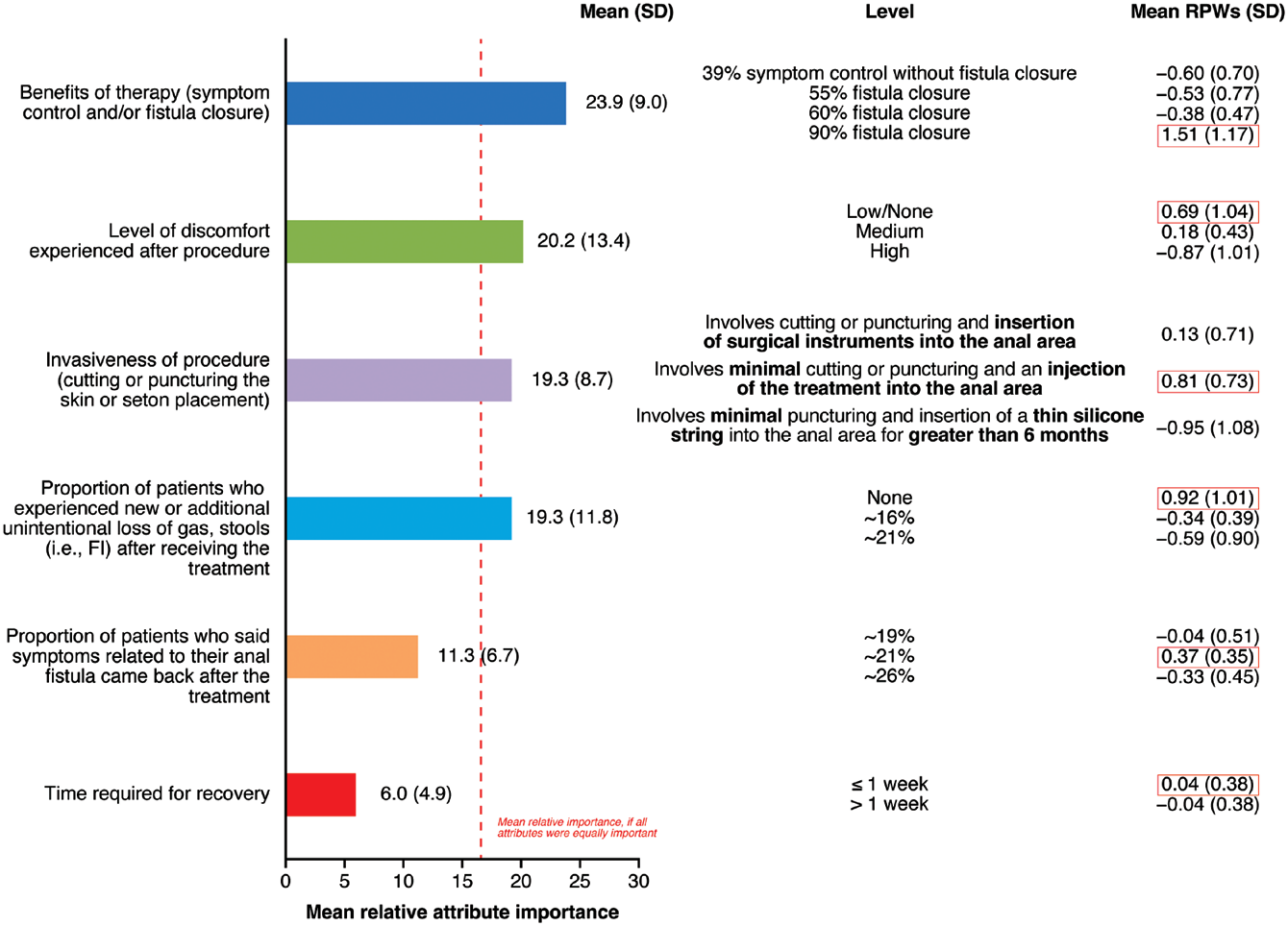
Relative importance of CPF-related procedure/surgery attributes and relative preference weights for specified attribute levels of CPF-related procedures/surgeries: patient preferences. Values next to bars represent mean (SD) importance scores. Rectangular boxes around RPWs indicate the highest level in each category. CPF, Crohn’s perianal fistulas; FI, fecal incontinence; RPW, relative preference weight; SD, standard deviation.

Overall, of the tested attributes, benefits of therapy (symptom control and/or fistula closure) and levels of postoperative discomfort were the most important attributes influencing patient choice in the treatment of CPF (Figure 2). Patient preferences were driven by treatments that result in high rates of fistula closure (90% closure), no FI, involve minimal cutting or puncturing, and an injection of the treatment into the anal area, and are associated with low/no postoperative discomfort (Figure 2).

#### DCE: HCP preferences

**Figure 3.**
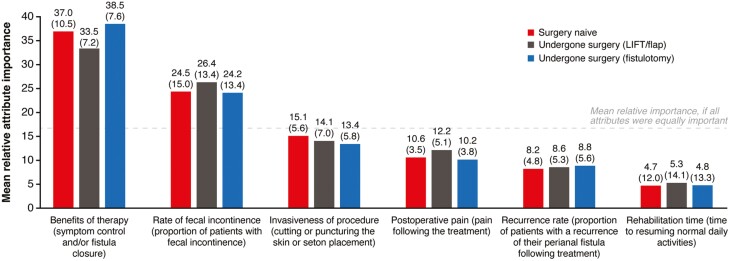
Relative importance of CPF-related procedure/surgery attributes: HCP preferences. Values above bars represent mean (SD) importance scores. CPF, Crohn’s perianal fistulas; HCP, healthcare professional; LIFT, ligation of the intersphincteric fistula tract; SD, standard deviation.

HCPs were asked to state their preference for hypothetical CPF-related procedures/surgeries based on the surgical experience of the patients they managed, categorized as: surgery-naive patients, patients with ligation of the intersphincteric fistula tract (LIFT)/flap procedures, and patients with fistulotomy. Overall, of the tested attributes, benefits of therapy (symptom control and/or fistula closure) and rates of FI were the most important attributes influencing HCP choice in the treatment of patients with CPF, irrespective of whether patients were surgery-naive or had undergone LIFT/flap or fistulotomy (Figure 3).

Preferences of HCPs for all the patients they managed were driven by treatments that result in high rates of fistula closure (90% closure) and no FI (Table 3). However, HCP preferences on procedure invasiveness differed for surgery-naive patients versus those who have undergone LIFT/flap procedures or fistulotomy (*P* < .001 for all levels). For surgery-naive patients, HCPs indicated a stronger preference for procedures involving minimal puncturing and insertion of a thin silicone string into the anal area for longer than 6 months compared with other options. In contrast, for patients who have undergone LIFT/flap procedures or fistulotomy, HCPs indicated a stronger preference for procedures involving minimal cutting or puncturing and an injection of the treatment into the anal area compared with other options (Table 3).

**Table 3.** Mean relative preference weights for specified attribute levels of CPF-related procedures/surgeries: HCP preferences

	Mean RPWs (SD)			
Attribute	Levels	Surgery-naive	Surgery post LIFT	Surgery post fistulotomy
Benefits of therapy (symptom control and/or fistula closure)	39% symptom control without fistula closure	–2.25 (1.00)	–1.49 (1.04)	–1.97 (1.06)
	55% fistula closure	–0.12 (0.63)	–0.73 (0.63)	–0.62 (0.58)
	60% fistula closure	0.22 (0.36)	–0.02 (0.43)	0.06 (0.48)
	90% fistula closure	2.15 (1.49)	2.23 (1.68)	2.54 (1.76)
Rate of FI (proportion of patients with FI)	None	1.46 (1.51)	1.63 (1.48)	1.53 (1.39)
	~16%	–0.54 (0.52)	–0.71 (0.58)	–0.62 (0.66)
	~21%	–0.92 (1.17)	–0.92 (1.05)	–0.90 (0.93)
Invasiveness of procedure (cutting or puncturing the skin or seton placement)	Involves cutting or puncturing and **insertion of surgical instruments into the anal area**	–0.51 (0.62)	0.03 (0.55)	–0.23 (0.57)
	Involves **minimal** cutting or puncturing and an **injection of the treatment into the anal area**	–0.03 (0.57)	0.46 (0.62)	0.24 (0.60)
	Involves **minimal** puncturing and insertion of a **thin silicone string** into the anal area for **longer than 6 months**	0.54 (0.69)	–0.49 (0.75)	–0.01 (0.78)
Postoperative pain (pain after the treatment)	Low/None	0.43 (0.47)	0.34 (0.50)	0.34 (0.41)
	Medium	0.06 (0.37)	0.10 (0.48)	0.07 (0.39)
	High	–0.48 (0.61)	–0.44 (0.54)	–0.41 (0.56)
Recurrence rate (proportion of patients with a recurrence of their perianal fistula after treatment)	~19%	0.20 (0.46)	0.13 (0.41)	0.06 (0.42)
	~21%	–0.01 (0.48)	–0.03 (0.44)	–0.07 (0.41)
	~26%	–0.19 (0.36)	–0.11 (0.42)	0.01 (0.46)
Rehabilitation time (time to resuming normal daily activities)	≤1 week	0.21 (0.27)	0.21 (0.28)	0.15 (0.29)
	>1 week	–0.21 (0.27)	–0.21 (0.28)	–0.15 (0.29)

DCE findings are reported overall for all GEs/CRSs (*N* = 137).

Abbreviations: CPF, Crohn’s perianal fistulas; CRS, colorectal surgeon; DCE, discrete choice experiment; FI, fecal incontinence; GE, gastroenterologist; HCP, healthcare professional; LIFT, ligation of the intersphincteric fistula tract; RPW, relative preference weight; SD, standard deviation.

#### DCE: Patient versus HCP preferences

**Figure 4.**
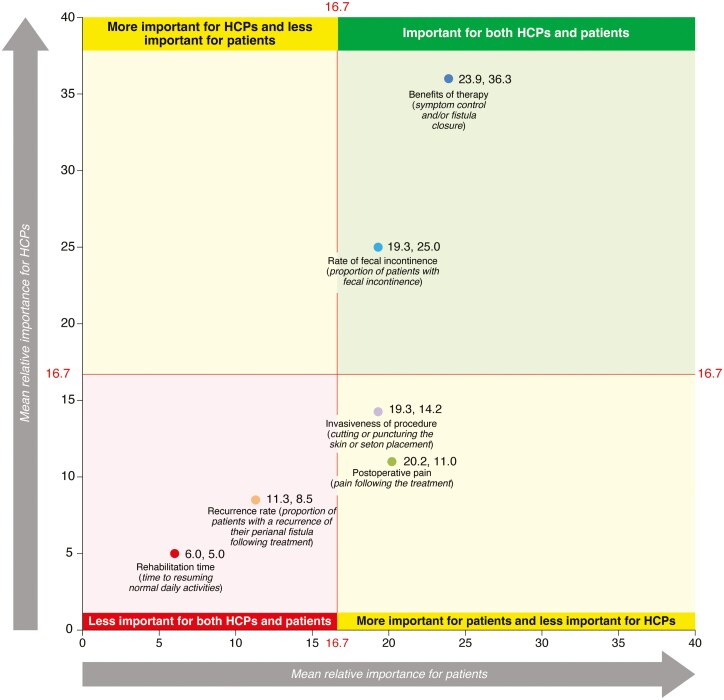
Comparison of patient and HCP perspectives on the importance of CPF-related procedure/surgery attributes. The horizontal axis represents mean relative importance for patients while making treatment decisions. The vertical axis represents mean relative importance for HCPs while making treatment decisions. Hence, the first number for each attribute in each quadrant is the mean score for patients and the second number is the mean score for HCPs. The overall mean relative importance scores for HCPs include their scores for surgery-naive and surgery experienced (LIFT and fistulotomy) patients. In total, 100 points were allocated to each product attribute from the DCE based on its importance when making CPF treatment decisions for both HCPs and patients. For the six attributes, if all were deemed equally important, then each would receive a point allocation of **16.7**. Therefore, attributes perceived as important to respondents had a score of **≥16.7**. CPF, Crohn’s perianal fistulas; DCE, discrete choice experiment; HCP, healthcare professional; LIFT, ligation of the intersphincteric fistula tract.

Both patients and HCPs considered benefits of therapy (symptom control and/or fistula closure) and rate of FI as important factors (relative importance score ≥16.7) during treatment consultations. Postoperative pain and invasiveness of procedure were considered more important by patients compared with HCPs. Rate of fistula recurrence and rehabilitation time were considered as the least important factors influencing treatment preferences by both patients and HCPs (Figure 4).

## Discussion

This is the first known DCE to assess the preferences of patients and HCPs in the USA for CPF-related procedures/surgeries. This study aimed to assess and compare patient and HCP preferences to better inform CPF management. Furthermore, as perianal disease often requires both medical and surgical interventions, necessitating a multidisciplinary approach that might not be fully understood by most patients, findings from this study could also help improve patient understanding of CPF management.

Overall, patient preferences in this US study are similar to those in a previous cross-sectional, observational study (Karki et al., 2023) conducted in Australia, Canada, France, Germany, Japan, Spain, and the UK, where a DCE was used to assess the treatment preferences of patients with CPF who had undergone perianal fistula-related surgery versus those who had not.^21^ Karki et al. showed that postoperative discomfort and rates of fistula healing were the first and second most important CPF treatment attributes to patients, respectively. In this US study, benefits of therapy (symptom control and/or fistula closure) were the most important treatment attribute and postoperative discomfort was the second most important attribute to patients. A difference in order of preference of the top 2 most important treatment attributes to patients between the multicountry and US DCEs may be due to differing methodology (as some of the treatment attribute wording differed between the studies: “symptom control/fistula closure” vs. “fistula healing”). Furthermore, differences in the study populations, and variations in CPF guidelines and perspectives between countries and regions, may influence the DCE outcomes. A numerically higher proportion of patients in the multicountry study had undergone long-term seton placement, anal fistula plug, fibrin glue, and LIFT compared with patients in this US study (data presented at the European Crohn’s and Colitis Organisation [ECCO] 2022 virtual congress).^22^ In addition, patients who participated in the multicountry study had higher treatment failure rates after endorectal/anal advancement flap, fibrin glue, and anal fistula plug than patients in this US study (data presented at ECCO 2022 virtual congress).^22^ Notably, the efficacy endpoint in the multicountry study was shown as “fistula healing”, which allowed setons to have a similar effect on the model as any other treatment option (eg, LIFT or endorectal/anal advancement flap).^21^ In contrast, the DCE model in the US study used the term “symptom control and/or fistula closure” where “symptom control” is a distinct benefit endpoint for setons. This difference may have cordoned off the effect on seton efficacy and resulted in the desire for symptom control driving a stronger preference for “symptom control and/or fistula closure” in the US study compared with “fistula healing” in the multicountry study. Finally, the most frequent complication experienced postsurgery by the patients in the multicountry study was fever/infection, while worsening of pain/difficulty with bowel movements was the most frequent postsurgery complication experienced by patients in this US study, which may influence patients’ perceived importance of postoperative discomfort as a treatment attribute in the US study.^21^

In this study, treatment preferences of HCPs for all the patients with CPF they managed were driven by treatments that result in high rates of fistula closure and lack of FI, irrespective of whether patients were surgery-naive or had undergone LIFT/flap or fistulotomy. However, HCP preferences on procedure invasiveness differed for surgery-naive patients versus those who have undergone LIFT/flap procedures or fistulotomy, with a less invasive approach preferred for surgery naïve patients.

Although patients and HCPs both rated benefits of therapy (symptom control and/or fistula closure) as the most important CPF-related procedure/surgery attribute, there were some significant differences in the preferences for certain procedure/surgery attributes between patients and HCPs; for example, postoperative pain and invasiveness of procedure were considered more important by patients than HCPs.

A previous study by Wong et al. showed that there are differences in patient and HCP preferences, specifically for HRQoL factors associated with anal fistulas and their treatment, which should be considered during the treatment decision-making.^10^ Wong et al. reported that both patients and HCPs rated pain, continence, and leakage as the most important HRQoL factors influencing treatment decisions. However, patients also included psychological health and independent activity in the top 5 most important HRQoL factors, whereas HCPs included cure and sepsis in their top 5.^10^

As there are some differences between patient and HCP perspectives on the attributes driving treatment decisions, an MDT approach to treatment decision-making that includes the patient’s voice is important to help HCPs understand which outcomes matter most to patients and would improve patient HRQoL. These differing perspectives of patients and HCPs should be considered when making treatment decisions and should be highlighted in HCP medical education.

This study has some limitations that should be acknowledged, including being subject to selection bias (eg, patients with more severe symptoms or treatment failures may be more likely to respond to questionnaires related to these topics), unintentional and nonresponse bias, question order bias, inaccuracies associated with self-reported measures, unconfirmed internal and external validity, and missing data. These limitations were partly mitigated by using pretest telephone interviews (to check the questions were fully understood by patients and HCPs), a web-enabled questionnaire (to minimize social desirability bias), and by limiting the recall period for clinical characteristics to 12 months or less. Inaccuracies associated with self-reported measures may indeed explain the unexpected treatment failure rates reported in this study; for example, failure rates for fistula plug were substantially lower than those of long-term seton placement (although it is worth noting, this study was not designed to offer epidemiological or pharmacoepidemiological insights and that treatment/management history was simply captured in an effort to contextualize the DCE results). In addition, the “benefits of therapy” attribute combined fistula closure and symptom control, making it hard to discern which of these 2 attributes were more important to patients and HCPs. Therefore, further research is required to evaluate patient and HCP preferences on individual treatment benefits. Furthermore, HCP demographics, such as years in active clinical practice or number of patients with CPF personally managed by each HCP, could be confounding variables in the responses. The number of HCPs in this survey was not large enough to allow a robust multivariable analysis of such potential confounders. Finally, the study did not include GEs/CRSs who may treat a lower number of patients with CPF than required for study eligibility or HCPs of different specialties, such as general surgeons, primary care physicians, nurse practitioners, or physician assistants.

## Conclusions

This study gained the perspectives of both patients and HCPs, providing holistic information to support treatment decisions in the management of CPF. Benefits of therapy (symptom control and/or fistula closure) were rated as the most important treatment attribute of CPF-related procedures/surgeries by both patients and HCPs in the USA. However, there were some differences in patient and HCP preferences for procedure/surgery attributes, and it is therefore important to consider both perspectives in the management of CPF. Findings from this study indicate that patients with CPF care about both the procedure experience and the procedure outcomes, while HCPs are more focused on the procedure outcomes. The treatment attributes identified as important to patients and HCPs could be considered as clinical endpoints when assessing the effectiveness of CPF treatments.

## Supplementary Material

otae076_suppl_Supplementary_Figure

## Data Availability

The datasets used and/or analyzed during the current study are available from the corresponding author on reasonable request.
