# Supplementary material for: Patient and Healthcare Professional Perspectives on Crohn’s Perianal Fistula Treatment: Results From a Discrete Choice Experiment
Source: Crohns Colitis 360. 2025 Jan 4;7(1):otae076. doi: 10.1093/crocol/otae076 (PMC11711682; doi:10.1093/crocol/otae076)
Supplement: otae076_suppl_Supplementary_Figure [file otae076_suppl_supplementary_figure.docx]

# SUPPLEMENTARY MATERIAL

Supplement to: ‘Patient and Healthcare Professional Perspectives on Crohn’s Perianal Fistula Treatment: Results From a Discrete Choice Experiment’.

**Supplementary Figure 1.** CPF-related procedure/surgery attributes and levels used to generate hypothetical surgical treatment options in the DCE to determine HCP preferences

**
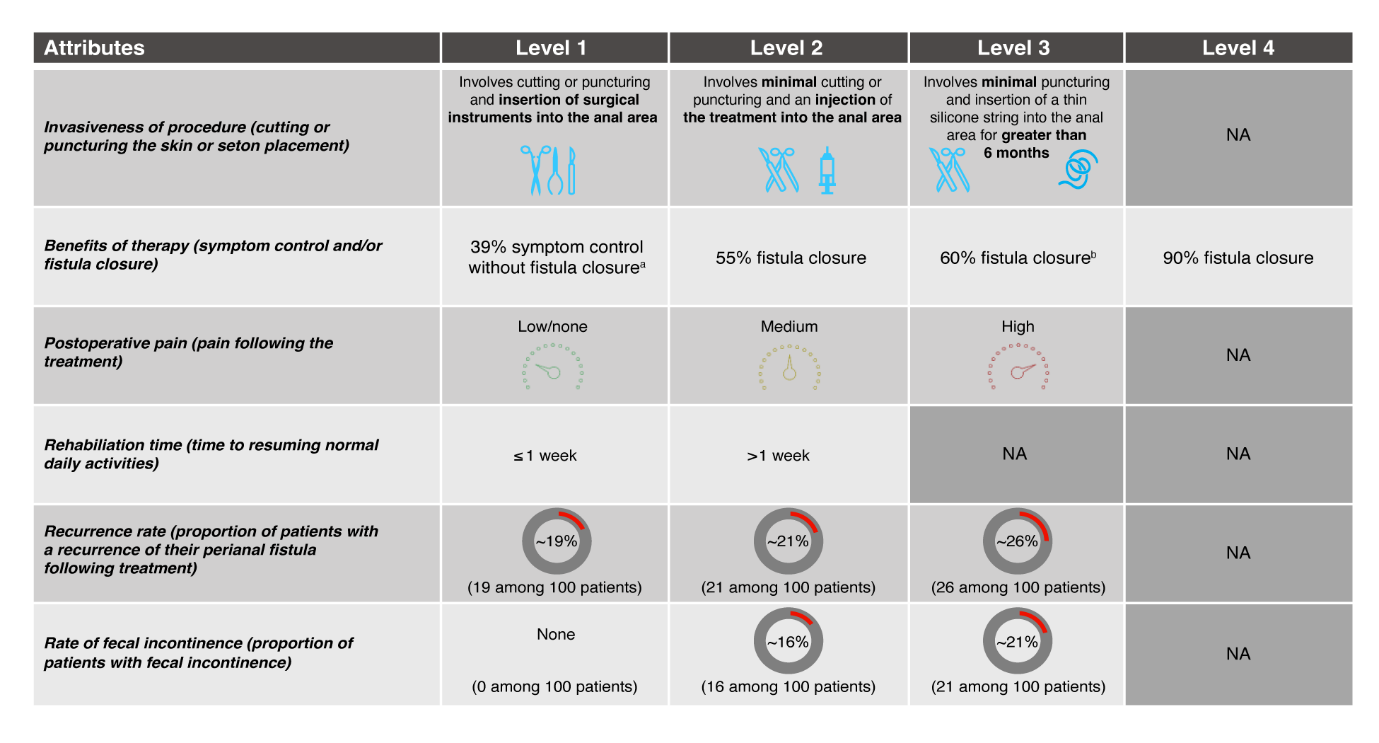
**

^a^This level was only shown with Attribute 1, Level 3; ^b^No level with fistula closure was shown with seton level above.

CPF, Crohn’s perianal fistulas; DCE, discrete choice experiment; HCP, healthcare professional; NA, not applicable.
